# Supplementary material for: The role of exosomal miRNA-125b derived from colon cancer-associated fibroblasts in skeletal muscle cachexia
Source: PLoS One. 2026 Feb 20;21(2):e0342052. doi: 10.1371/journal.pone.0342052 (PMC12923045; doi:10.1371/journal.pone.0342052)
Supplement: S2 Fig — (PDF) [file pone.0342052.s002.pdf]

Figure 1C

Figure 1C

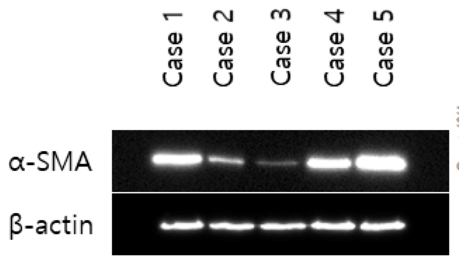

Alpha-SMA (42kDa)

43kDa

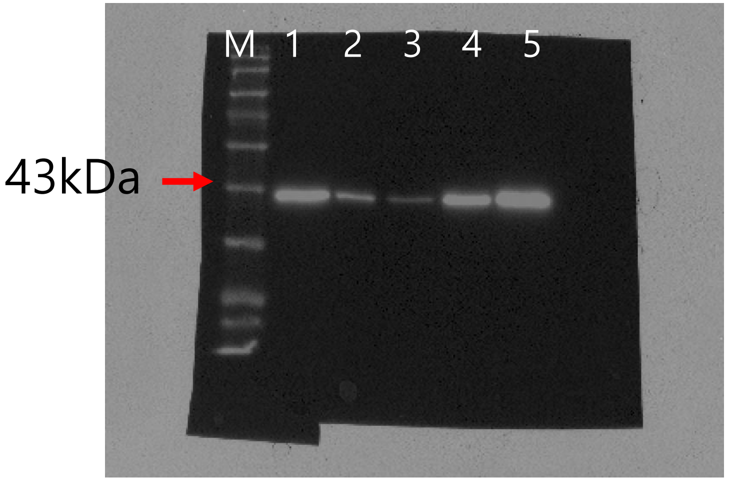

Beta-actin(45kDa)

43kDa

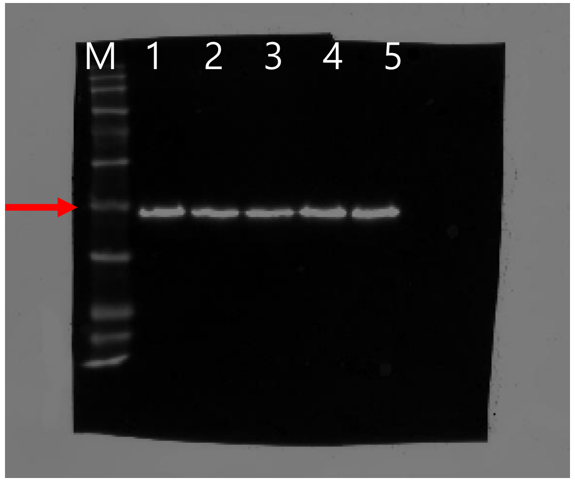

M. Marker
1. Case 1
2. Case 2
3. Case 3
4. Case 4
5. Case 5

As requested, we have provided the uncropped original Western blot images. These blots were performed on PVDF membranes and visualized using the Bio-Rad ChemiDoc Imaging System. For molecular weight reference, we used the 10-250 kDa Xpert Prestained Protein Marker (P8502-050, genDEPOT, USA).

Figure 2B

Case 1

Figure 2B

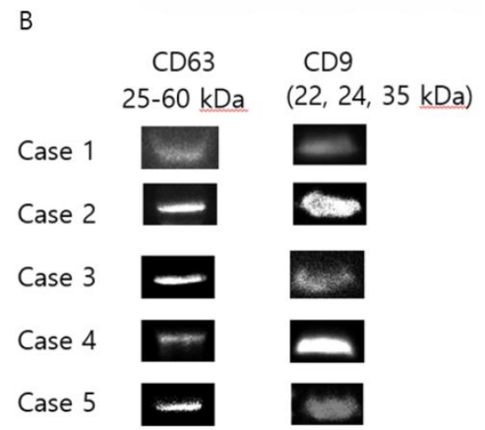

CD63
25-60 kDa

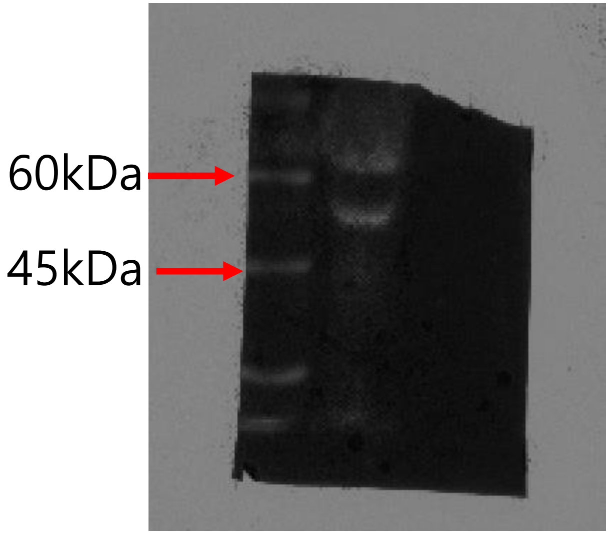

CD9
(22, 24, 35 kDa)

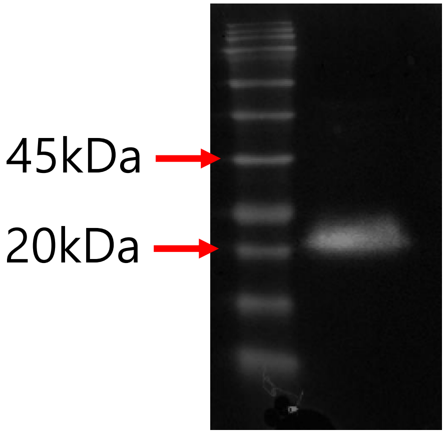

As requested, we have provided the uncropped original Western blot images. These blots were performed on PVDF membranes and visualized using the Bio-Rad ChemiDoc Imaging System. For molecular weight reference, we used the 10–245 kDa Raon Multi-color Protein Marker (PB700, SMObio, Taiwan).

Case 2

Figure 2B

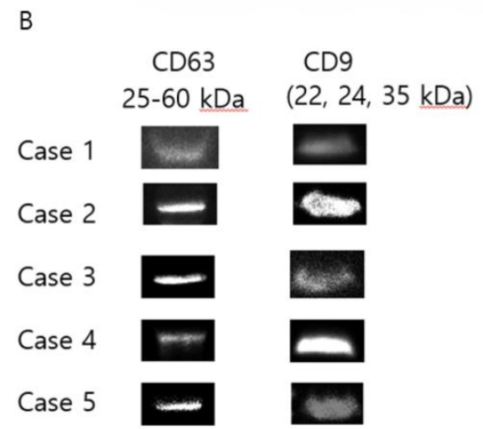

CD63
25-60 kDa

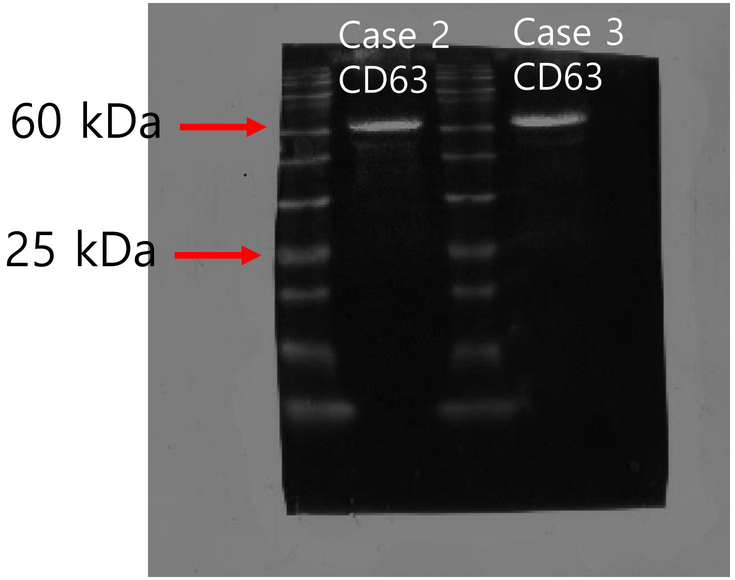

CD9
(22, 24, 35 kDa)

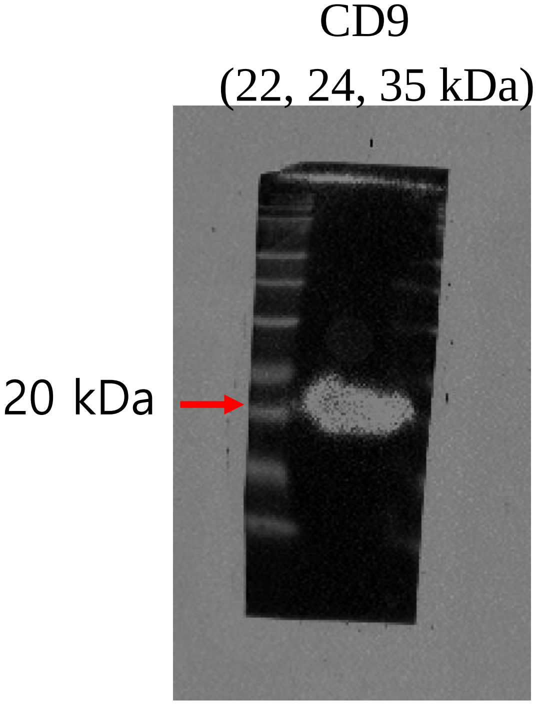

As requested, we have provided the uncropped original Western blot images. These blots were performed on PVDF membranes and visualized using the Bio-Rad ChemiDoc Imaging System. For molecular weight reference, we used the 10–245 kDa Raon Multi-color Protein Marker (PB700, SMObio, Taiwan).

Case 3

Figure 2B

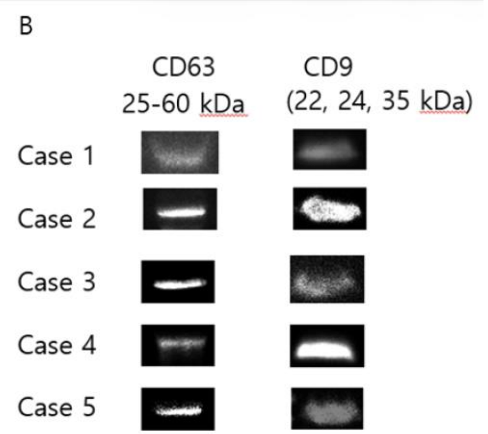

CD63

25-60 kDa

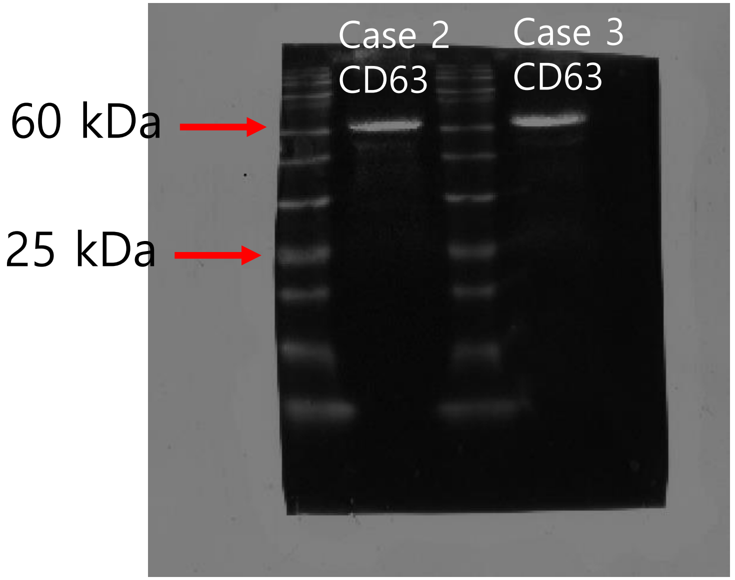

CD9

(22, 24, 35 kDa)

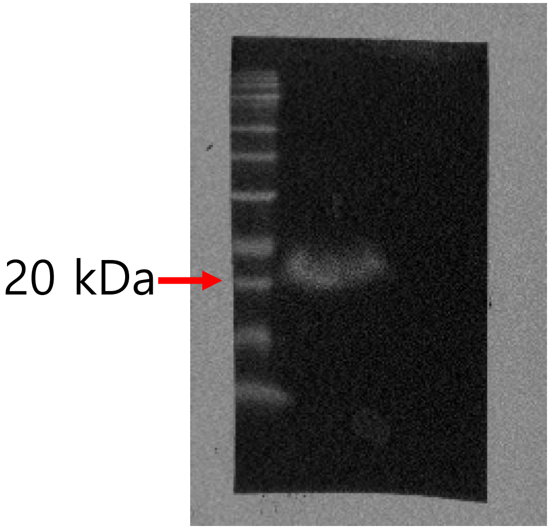

As requested, we have provided the uncropped original Western blot images. These blots were performed on PVDF membranes and visualized using the Bio-Rad ChemiDoc Imaging System. For molecular weight reference, we used the 10–245 kDa Raon Multi-color Protein Marker (PB700, SMObio, Taiwan).

Case 4

Figure 2B

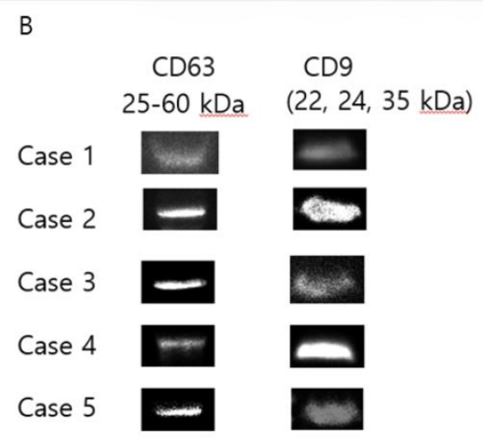

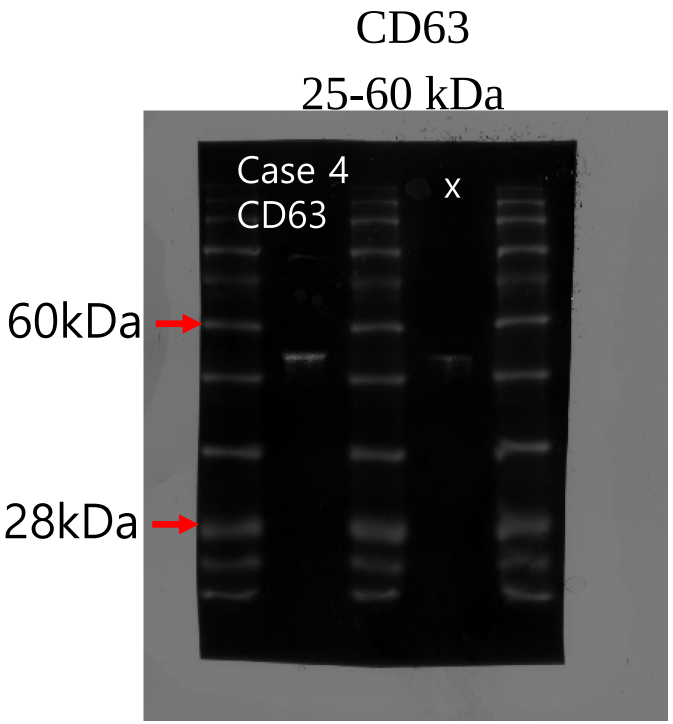

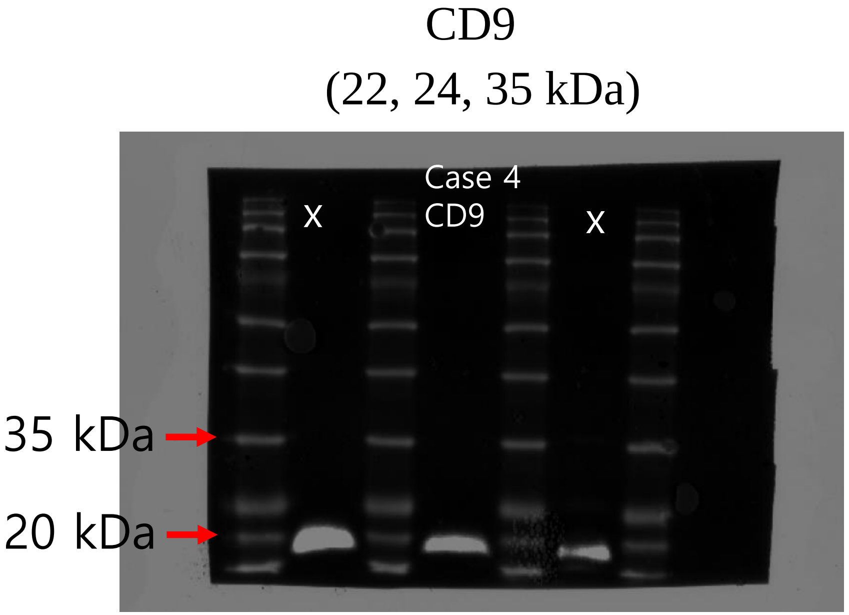

As requested, we have provided the uncropped original Western blot images. These blots were performed on PVDF membranes and visualized using the Bio-Rad ChemiDoc Imaging System. For molecular weight reference, we used the 10-250 kDa Xpert Prestained Protein Marker (P8502-050, genDEPOT, USA).

Case 5

CD63
25-60 kDa

CD9
(22, 24, 35 kDa)

Figure 2B

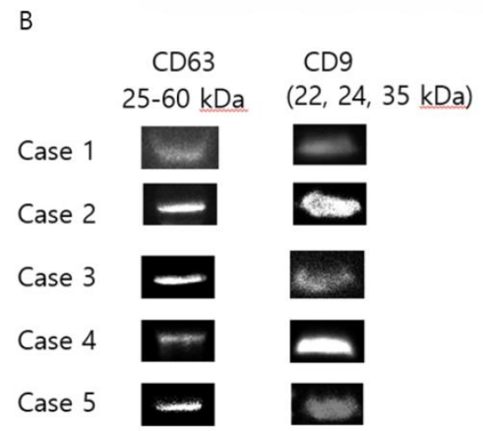

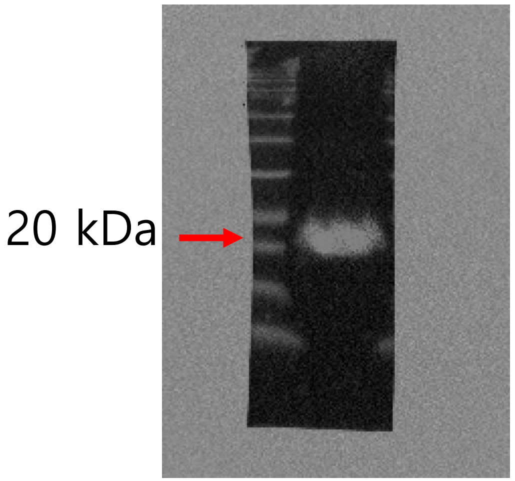

As requested, we have provided the uncropped original Western blot images. These blots were performed on PVDF membranes and visualized using the Bio-Rad ChemiDoc Imaging System. For molecular weight reference, we used the 10–245 kDa Raon Multi-color Protein Marker (PB700, SMObio, Taiwan).
