# Supplementary material for: The role of exosomal miRNA-125b derived from colon cancer-associated fibroblasts in skeletal muscle cachexia
Source: PLoS One. 2026 Feb 20;21(2):e0342052. doi: 10.1371/journal.pone.0342052 (PMC12923045; doi:10.1371/journal.pone.0342052)
Supplement: Attachment — Submitted filename: Point-to-point_responses.docx [file pone.0342052.s003.docx]

Journal Requirements

1. When submitting your revision, we need you to address these additional requirements.

R1. We have adhered to the journal guidelines as closely as possible. Please let us know if any further changes are necessary.

2. Journal Requirements - Funding statement

R2. Thank you for pointing this out. Kindly provide this sentence in the online submission system on our behalf:

*“**The funders had no role in study design, data collection and analysis, decision to publish, or preparation of the manuscript.”*

3. Journal Requirements - Data availability

R3. In addition to the data already presented in the figures and main text, we have attached a file containing the numerical values underlying all graphs (including myosin diameter measurements and cell viability data) as “Supporting Information File 1. Raw data for figures.”

4. PLOS requires an ORCID ID for the corresponding author

R4. We re-checked that the corresponding author’s ORCID ID has been supplied in the Editorial Manager.

5. Journal Requirements – Original blot images

R5. We appreciate the reminder regarding blot and gel reporting. As requested, we have provided the uncropped original western blot images (Figures 1C, 2B) and uploaded them as “Supporting Information File 2. Original Images for Blots.” The figure panels in the main manuscript have been checked and revised if needed to fully adhere to the PLOS ONE blot and gel guidelines.

We regret to inform you that the original western blot data for CD63 in Case 5 are no longer available. The experiment was performed approximately one year ago, and we made extensive efforts to retrieve the raw data, including checking the internal storage of the instrument used for image acquisition. However, because the instrument is shared among various institutions, stored data are periodically deleted by the system administrator as part of the routine maintenance. Therefore, the original files for this experiment, including the raw data for this specific blot, could not be located. Repeating the same experiment to compensate for the missing data was also considered. However, the relevant samples are no longer available. We respectfully ask for your understanding of these limitations.

6, 7. Journal Requirements – Reference list

R6,7. We have checked all references again for accuracy. No article was retracted. Following the comments of reviewer 1, we searched for the most recent data in relevant papers. Unfortunately, it was difficult to find studies on miRNAs in cachexia, particularly on CAF-derived exosomes.

Reviewer #1

C1. Inadequate Research Gap Justification

The introduction is overly broad and fails to specify the precise knowledge gap this study fills or why it is necessary. It requires more solid placement in the body of current literature.

R1. We thank the reviewer for this important comment. We agree that the research gap needs to be described more explicitly. Previous studies on cancer cachexia have focused on exosomes derived directly from tumor cells or from circulating biofluids, whereas the role of exosomes derived specifically from cancer-associated fibroblasts (CAFs) has rarely been investigated. We aimed at addressing this gap by examining the effect of CAF-derived exosomes on cachexia.

We have therefore revised the Introduction to better position our study within the current literature.

*“**Although several studies have implicated tumor cell-derived or circulating exosomes in the development of cancer-associated cachexia, the specific contribution of exosomes released by CAFs remains largely unexplored. Especially, whether CAF-derived exosomal miRNAs directly influence skeletal muscle atrophy has not been clarified. Therefore, in this study, we aimed to investigate the effects of CAF-derived exosomes from colon cancer tissues on skeletal muscle cells, with a particular focus on exosomal miRNAs.”*

C2. The second is methodological transparency.

A completely replicable process is required by PLOS ONE. A few details are lacking: the recruitment process and sample size calculation are imprecise, and the precise inclusion/exclusion criteria are not properly specified. The way statistical approaches handle missing data and correct for confounding is not explained.

R2. We appreciate this helpful comment. Because this is an exploratory in vitro study using primary CAFs and not a clinical outcomes study, no formal sample size calculation was performed; instead, we used all eligible cases that met the criteria during the study period (n = 5), which we now explicitly state. Moreover, since our study focused on cellular phenotypes rather than clinical outcomes, no adjustment for clinical confounders was required. The proportion of missing data was very small, and observations with missing values were excluded from the analyses (Additional file attached, Supporting Information File 1. Raw data for figures). The inclusion and exclusion criteria and consent process have already been described in the “Patients and samples” section of the manuscript. We would be happy to provide additional information if required.

C3. Results Presentation: Some findings are descriptive and lack critical analysis.

According to PLOS ONE statistical reporting guidelines, p-values should be accompanied with confidence ranges.

R3. We thank the reviewer for this valuable suggestion. In accordance with the statistical guidelines of PLOS ONE, we recognized all quantitative datasets should present 95% confidence intervals (CI) alongside p-values. However, because we performed multiple experimental comparisons across several figures, we considered reporting confidence intervals for all statistical results impractical. Moreover, confidence intervals are not routinely reported in this field, in which statistical significance is typically presented using the mean ± SD/SEM and p-values. We are willing to provide additional statistical output, including confidence intervals for key comparisons, upon request. We believe that the current presentation adequately conveys the robustness of the experimental findings while remaining consistent with common reporting practices in experimental research.

C4. Discussion

Excessively detailed and devoid of critical comparison with the most recent research. Given the cross-sectional/observational study methodology, a number of statements are exaggerated. The limitations section is inadequate and needs to address potential biases (generalisability, reporting, and selection) in greater detail.

R4. We thank the reviewer for this important comment. We agree that incorporating recent literature is essential for contextualizing our findings. In the initial version of the manuscript, we attempted to include recent studies regarding cancer cachexia and miRNA-mediated muscle atrophy. However, the number of studies specifically addressing miRNA derived from CAF-derived exosomes remains extremely limited. While several reports describe tumor-cell or circulating exosomal miRNAs in cachexia, we were unable to identify prior work demonstrating a cachectic effect mediated by miRNAs originating from CAF-derived vesicles, which highlights the novelty of our approach.

We further agree that some phrases in the Discussion were stronger than appropriate for an observational in vitro study. Similar feedback was also raised by reviewer 2, and we have therefore carefully reviewed the entire manuscript to ensure that interpretations are presented cautiously. We have toned down statements throughout the Discussion section.

Additionally, the limitations section has been extensively revised, also taking into account the comments of reviewer 2.

*“This study has a few limitations. First, the use of CAF samples from a single institution may introduce selection or reporting bias, and the relatively small sample size limits generalizability. Future studies involving larger stromal sample sets are necessary to validate reproducibility. Second, the interpretation of muscle atrophy was primarily based on morphological thinning of myosin fibers, and functional biomarkers of proteolysis such as atrogin-1, muscle RING-finger protein-1, myoblast determination protein 1, or myogenin were not quantified. Therefore, we cannot fully exclude the possibility that the observed phenotype reflects cytoskeletal remodeling rather than definitive cachexia-associated proteolytic activation. Third, downstream mechanistic pathways regulated by miR-125b were not characterized, and signaling interactions between CAF-derived vesicles and muscle cell proteostasis remain undefined. Fourth, miRNA sequencing was performed on a single sample, which may restrict the robustness of the miRNA profile. Moreover, we did not evaluate in vivo muscle wasting or systemic metabolic consequences; thus, the biological relevance of CAF-derived exosomal miR-125b requires further confirmation in animal models. Despite these limitations, our results provide novel evidence that CAF-derived exosomal miR-125b may contribute to skeletal muscle atrophy and suggest a stromal miRNA axis in cancer cachexia that has not been previously clarified.”*

C5. Conclusion: Overly conclusive; has to be careful and closely linked to the results of the study.

R5. We thank the reviewer for pointing this out. We have revised the conclusion to more cautiously reflect the scope of our findings.

C6. Formatting and Journal Style Issues

1. Abstract: Structured abstracts (Background, Methods, Results, Conclusion) are required by PLOS ONE. The abstract as it stands now is repetitious and narrative.

2. References: The formatting is not in accordance with PLOS ONE policy, which requires a numbered citation style based in Vancouver.

o A number of the references are out of date; there are no fresh sources from 2022–2024.

3. Tables and Figures: o A few figure legends lack details and make it impossible to understand them without consulting the main text.

o There is excessive narrative material in the tables, which deviates from journal formatting guidelines.

4. Language and Grammar o Several typographical and grammatical mistakes.

o Uncomfortable wording (such as "patients was," "can able to") makes it harder to read.

o For clarity and flow, extensive English editing is required.

R6. Formatting, Figures, and Language

We thank the reviewer for these helpful suggestions.

1. Our paper is an experimental paper, and previous papers published in PLOS One have a one-paragraph abstract. Moreover, the journal guidelines do not specify the need for subsections in the abstract. We would thus like to refrain from revising anything in this respect.

2. We used Vancouver style for citations. We have double-checked the in-text citations and reference list and performed revisions as needed.

3. Figure legends have been revised so that they are self-explanatory.

4. We have proofread the entire manuscript and ensured that the text is error-free and reads smoothly.

Reviewer #2:

C0. This manuscript entitled “The Role of exosomal miRNA-125b derived from colon cancer-associated fibroblasts in skeletal muscle cachexia” explores the contribution of CAF-derived exosomal miR-125b to skeletal muscle atrophy. The study is interesting and addresses an important gap in understanding how stromal components of the tumor microenvironment influence cancer-associated cachexia. Overall, the manuscript is clearly written and logically structured, but several issues related to experimental robustness, quantitative analysis, and mechanistic depth should be addressed before acceptance.

R0. We sincerely thank the reviewer for the thoughtful and insightful evaluation. We appreciate the recognition of the novelty and relevance of investigating CAF-derived exosomes in cancer cachexia. We have carefully revised the manuscript to address all comments, expand experimental validity, strengthen mechanistic interpretation, and improve statistical rigor.

C1. miRNA sequencing appears to have been performed on a single CAF-derived exosome sample. This significantly limits the robustness of the findings. At minimum, please validate the enrichment of miR-125b across all CAF isolates using qRT-PCR and acknowledge this limitation explicitly in the Methods and Discussion.

R1. We agree with the reviewer that validation across multiple CAF-derived exosomes is essential for robustness. Unfortunately, CAF-derived exosomes are currently unavailable, preventing further experiments. Even in the initial experiments, extracting sufficient exosomes for miRNA sequencing was time-consuming and challenging, and for practical reasons, only a single sample was available. These limitations have been described in the "Limitations" section.

*“Fourth, miRNA sequencing was performed on a single sample, which may restrict the robustness of the miRNA profile.”*

C2. The conclusions are primarily based on morphological observations (myosin diameter). Without functional markers (e.g., atrogin-1, MuRF-1, MyoD, Myogenin, or IGF-1 pathway readouts), it remains uncertain whether the observed changes truly represent cachexia-related atrophy rather than a general cytoskeletal alteration. Incorporating at least one molecular validation experiment or a more nuanced discussion of this limitation is strongly encouraged.

R2. We thank the reviewer for raising this important point. We made every effort to perform additional validation experiments using western blot analysis of MyoD (13812; Cell Signaling Technology, Danvers, MA, USA) and Atrogin‑1 (AP2041, Antibodies Incorporated, Davis, CA, USA) in our human skeletal muscle cell cachexia model. However, we encountered significant technical limitations, including non‑specific bands and high background signals, with the currently available antibodies. This prevented us from obtaining reliable data within the given time frame. We have attached the experimental results below.

We have also revised the limitations section according to the reviewer’s comment.

*“Second, the interpretation of muscle atrophy was primarily based on morphological thinning of myosin fibers, and functional biomarkers of proteolysis such as atrogin-1, muscle RING-finger protein-1, myoblast determination protein 1, or myogenin were not quantified. Therefore, we cannot fully exclude the possibility that the observed phenotype reflects cytoskeletal remodeling rather than definitive cachexia-associated proteolytic activation.”*

C3. The statistical analysis relies exclusively on Student’s t-tests, even in cases where more than two groups were compared (e.g., Figs 4, 7, 8, 9, 10). Please confirm that all data met the assumptions of parametric testing or use one-way ANOVA with post-hoc correction. Figure legends should include n values, the type of error bars (SD or SEM), and exact p-values.

R3. We appreciate this statistical recommendation and fully agree with the comment. All experiments involving more than two conditions were re-analyzed using one-way ANOVA with Tukey’s post hoc test. Figure legends now report ANOVA statistics, exact p-values, n-values, and error bars (mean ± SD/SEM). We have also revised Figures 9 and 10 according to our re-analysis.

The statistics section has been modified as follows.

“*Data are expressed as mean ± standard deviation (SD) or standard error of the mean (SEM). Statistical significance was determined using one-way analysis of variance followed by Tukey’s post hoc test (* P < 0.05,** P < 0.01, and *** < 0.001).”*

C4. The reported mean particle size of ~28 nm is notably smaller than typical exosomes (50–150 nm). This discrepancy raises concern about the accuracy of NTA measurements or sample purity. Please re-examine the data or provide an explanation (e.g., detection threshold differences or aggregation).

R4. We thank the reviewer for this insightful comment. We also had concerns regarding the relatively small mean particle size (~28 nm) in the NTA analysis. NTA measurement was performed approximately 6 months after initial exosome isolation to confirm the adequacy of the exosome extraction process. We suspect that delayed processing and long-term storage may have influenced vesicle stability and size distribution.

In response to the reviewer’s suggestion, we have revised the figure legend to explicitly state that NTA measurement was performed 6 months after exosome extraction. We believe that this clarification improves the transparency of our experimental timeline and helps explain the observed size profile.

*“**This analysis was conducted approximately 6 months after initial exosome extraction. Delayed processing may have contributed to the smaller mean particle size observed.”*

C5. The manuscript occasionally overstates causality. For example, asserting that miR-125b “directly induces muscle atrophy.” Given the correlative nature of the current data, the statement should be tempered and rephrased to reflect a potential association or preliminary mechanistic indication.

R5. We appreciate this comment. Reviewer 1 raised the same point. All statements suggesting causality have been toned down.

C6. Other comments:

- The discussion is generally well-written but repetitive. It should focus more on how CAF-derived miR-125b differs from tumor-cell-derived or serum exosomal miRNAs.

- The limitations section is appreciated but should include the lack of in vivo validation and absence of protein-level mechanistic studies.

R6. We have reduced repetitive passages in the Discussion, added new text comparing CAF-derived exosomal miRNAs with tumor-derived and serum-exosomal miRNAs, and expanded the limitations section to acknowledge the absence of in vivo validation and downstream target profiling.

*“Furthermore, the biological function and regulatory context of CAF-derived exosomal miR-125b may differ from those of tumor cell-derived or serum exosomal miRNAs. However, studies directly comparing these sources are extremely rare. Further research is required to clarify whether stromal-derived miRNAs exert cachexia-driving effects distinct from those of tumor-derived miRNAs or circulating vesicles.”*
